# Production of Lectins from Marine Algae: Current Status, Challenges, and Opportunities for Non-Destructive Extraction

**DOI:** 10.3390/md20020102

**Published:** 2022-01-26

**Authors:** Intan Mariana Maliki, Mailin Misson, Peik Lin Teoh, Kenneth Francis Rodrigues, Wilson Thau Lym Yong

**Affiliations:** Biotechnology Research Institute, Universiti Malaysia Sabah, Kota Kinabalu 88400, Sabah, Malaysia; imarianamaliki@gmail.com (I.M.M.); mailin@ums.edu.my (M.M.); peiklin@ums.edu.my (P.L.T.); kennethr@ums.edu.my (K.F.R.)

**Keywords:** algal lectin, culture media, large scale production, marine algae, non-destructive extraction

## Abstract

Marine algae are an excellent source of novel lectins. The isolation of lectins from marine algae expands the diversity in structure and carbohydrate specificities of lectins isolated from other sources. Marine algal lectins have been reported to have antiviral, antitumor, and antibacterial activity. Lectins are typically isolated from marine algae by grinding the algal tissue with liquid nitrogen and extracting with buffer and alcohol. While this method produces higher yields, it may not be sustainable for large-scale production, because a large amount of biomass is required to produce a minute amount of compound, and a significant amount of waste is generated during the extraction process. Therefore, non-destructive extraction using algal culture water could be used to ensure a continuous supply of lectins without exclusively disrupting the marine algae. This review discusses the traditional and recent advancements in algal lectin extraction methods over the last decade, as well as the steps required for large-scale production. The challenges and prospects of various extraction methods (destructive and non-destructive) are also discussed.

## 1. Introduction

Macrophytic marine algae, also known as seaweeds, are nonvascular, multicellular, photosynthetic aquatic plants that live in the coastal regions of ocean waters, typically in intertidal or submerged reef-like habitats [1]. Seaweeds are classified into three major groups based on their pigment color: Chlorophyta (green seaweed), Phaeophyceae (brown seaweed), and Rhodophyta (red seaweed) [2]. Several types of valuable proteins, such as peptides, enzymes, glycoproteins, lectins, and mycosporine-like amino acids, are abundant in seaweeds, accounting for up to 50% of dry weight [2].

A “lectin” is a protein that possesses the ability to agglutinate red blood cells with known sugar specificity. When the sugar specificity is unknown, the protein is referred to as “hemagglutinin” [3]. Terrestrial plant lectins are typically found in seeds and other tissues and organs such as roots, tubers, bulbs, bark, leaves, and flowers [4]. In 1966, Boyd and his associates [5] discovered lectin in 24 algae species off the coast of Puerto Rico, which was the first discovery of lectin in marine algae. Since then, numerous studies on marine algal lectin have been published. Lectins are a type of non-immune protein or glycoprotein that can bind to carbohydrates or glycol components on cell surfaces reversibly [6,7]. Lectins are primary metabolites found in marine algae that play a role in physiological functions, including reproduction under normal growth conditions [3,7].

Traditionally, lectin was extracted from marine algae using mechanical and enzymatic methods. These methods were discovered to be laborious and time-consuming [8]. Therefore, rapid and high-throughput methods for lectin extraction are required for large-scale and sustainable production. Several protein extraction methods have since been published, including the enzyme-assisted method (EAE), the ultrasound-assisted method (UAE), and the supercritical fluid extraction method (SFE). While these methods were successful in isolating bioactive compounds from marine algae [9,10,11,12], there were few reports on lectin extraction specifically. Until recently, a revolutionary non-destructive lectin extraction approach known as cold steeping infusion (CSI) has been published [13]. This method enables the extraction of algal lectins from culture media without damaging the marine algae, and allows the algae to revert to their metabolic state following extraction. Nevertheless, reports on large-scale production of marine algal bioactive compounds, particularly algal lectin, are scarce. Therefore, more research into the nature and biology of marine algae, and advancements in extraction technologies, are required to better understand the challenges in algal lectin extraction and achieve successful production at larger scales for medical, pharmaceutical, and biotechnological applications.

## 2. Marine Algae—A Vital Source of Lectins

Marine algae are renowned as a rich source of protein and are regarded as an alternative source of protein due to their high protein and amino acid content [14]. Apart from that, marine algae contain a high concentration of secondary metabolites, which can be converted into a variety of bioactive compounds with diverse biological activities. The protein content of various types of marine algae differs depending on their classification. Brown algae has a low protein content in dry matter basis (DMB) of around 24–166 g/kg, whereas green and red algae have higher protein contents of 32–352 g/kg and 64–376 g/kg, respectively [15]. However, these values can vary depending on the genus species, growing environment, and harvest time [16,17]. Table 1 shows the protein composition of various marine algae species on a dry matter basis.

Lectins are carbohydrate-binding proteins that are not enzymes or antibodies [24,25]. They can form a relatively strong complex due to their specific affinities to specific glycan structures [26,27]. Stillmark [28] first discovered lectins in the seeds of *Ricinus communis* in 1888, and he went on to be a pioneer in isolating lectins from plant seeds and roots, bacteria, algae, fungi, and mammalian cell membranes. Lectins have gained popularity in biological and biomedical research as the most flexible group of proteins with immense potential, as they play a significant role in cell–cell recognition and drug delivery [29]. Their ability to decipher glycocodes, and their carbohydrate-binding specificity, molecular structure, and biochemical properties, contribute to their high level of biological activity [29]. Lectins have been found in most classes and families of organisms studied thus far, indicating that they are widely distributed in a diverse range of organisms [24]. Marine algal lectins have a proteinaceous content comparable to terrestrial plants. In general, they have low molecular masses, no affinity for monosaccharides, divalent cation-independent hemagglutination, and a high specificity for complex oligosaccharides, often glycoproteins [24,30]. Marine algae have also been reported to have anti-cancer, antiviral, and antibacterial properties [31,32,33,34,35]. The majority of anti-cancer properties are activated by binding on cancer cell membranes, resulting in cytotoxicity, apoptosis, and tumor growth inhibition [31]. On the other hand, algal lectins have antiviral properties due to their specific binding properties to carbohydrate structures that allow them to inhibit virus replication via interactions with viral envelope proteins [36].

Marine algae are an excellent source of novel lectins, with examples being Griffithsin from the red alga *Griffithsia* [37], SfL-1 and SfL-2 from *Solieria filiformis* [32], and HRL40 from *Halimeda renschii* [38]. More research into the biotechnological functions of lectins has begun, including antiviral activity against the hepatitis C virus (HCV) [35], induced cell death in human cancer cells [34], inhibition of streptococcal adherence by algal lectin extracted from *Bryothamnion triquetrum* and *Bryothamnion seaforthii* [30], and mitogenic activity of mouse spleen T lymphocytes [39]. The emergence of the novel coronavirus SARS-CoV-2 in December 2019 has raised awareness about the importance of antiviral agents and therapeutic concepts in controlling the outbreak, as vaccination is not always readily available. Many studies have shown that lectins can prevent the influenza virus from entering host cells, making them a promising candidate [33,40,41,42]. Table 2 depicts various marine algae species, their corresponding lectins, and their actions such as anticancer, antivirus, and drug delivery. For example, algal lectin KAA-2 from *Kappaphycus alvarezii* has been found to inhibit influenza virus entry into mammalian cells by binding directly to high mannose (HM)-type *N*-glycans of the viral envelope hemagglutinin (HA) [33]. HRL40 lectin, isolated from green alga *Halimeda renschii*, was observed to prevent influenza virus (A/H3N2/Udorn/72) infection in NCI-H292 cells via high-affinity binding to the viral envelope hemagglutinin at a half-maximal effective dose (ED50) of 2.5 nM [38].

## 3. Challenges in Algal Lectin Extraction

Algal lectins and their extraction are a relatively understudied research area compared to mushrooms and higher plants such as kidney beans and wisteria plants. It is critical to have a high level of accessibility to the protein molecules in the cells to extract the protein successfully. The presence of cell wall mucilage, however, limits the efficiency of algal lectin extraction [2,20,58]. The presence of cell walls and intracellular polysaccharides causes high viscosity and ionic interaction, which impedes extraction [58]. Another factor that may influence the extraction process is the morphology of the marine algae themselves; for example, marine algae with tougher thallus may require more processing [59]. To maximize lectin yield from marine algae, raw biomass should be freeze dried prior to extraction or used fresh as soon as possible to avoid protein degradation [2].

Traditional methods for extracting algal protein include aqueous, acidic and alkaline methods, followed by several rounds of centrifugation and recovery using techniques such as ultrafiltration, precipitation, or chromatography [8,60]. Other methods, such as two-phase alkali and acid treatment, have also been demonstrated to be effective in extracting proteins from marine algae. With regards to algal lectin extraction specifically, some common traditional methods include mechanical (maceration, physical grinding with liquid nitrogen) and enzymatic procedures [8], and the majority of research to date has used the methods listed in Table 2.

Although these methods yielded promising results, there were some concerns, such as the possibility that traditional mechanical and enzymatic extraction methods may affect the extracted algal lectin due to the release of proteases from cytosolic vacuoles [61]. Aside from that, these methods are time-consuming and labor-intensive, requiring significant improvements in cell disruption and extraction processes. Conventional methods of algal protein extraction are both resource inefficient and environmentally unfriendly, as algae by-products are often discarded after processing [8]. When the process is scaled up, the problem becomes much larger, and the waste becomes even more pronounced. Hence, improvements in terms of time, cost, energy consumption, and health hazard management towards humans and the environment are required.

Marine algae as a source of lectin require a concentrated form of high-quality extracts for various downstream applications. Isolating and purifying lectin from marine algae has been accomplished primarily through manipulation of initial extraction methods, as other methods have not been explored further. The purification of novel lectins is also a challenge due to their unknown physicochemical properties. The final application, production scale, and initial extraction method should all be taken into account when selecting a lectin purification method [59].

## 4. Lectin Production from Algal Cell Suspension

Suspension culture, also known as cell suspension, is a useful method for providing materials for high-throughput studies such as plant secondary metabolite production, allowing a large number of cells to be simultaneously screened for desired traits and facilitating research on a physiologically and biochemically homogenous population of cells [62,63]. Cell suspension cultures have been used to produce lectins in higher plants, including peanut agglutinin from *Arachis hypogea* cotyledon-derived suspension cultures, DB58 lectin from *Dolichos biflorus* cell suspension cultures, and Aralin lectin from *Aralia elata* callus cultures [64,65,66,67,68]. Rorrer et al. [69,70] demonstrated that marine algal cell culture can be used to manufacture various bioactive metabolites; however, there are limited studies on the production of lectins specifically.

Chen [71] established a suspension culture from *Porphyra linearis* protoplasts in 1989, making it one of the earliest reports of successful cell suspension culture of marine algae. This culture was maintained and cultured for four years without the formation of organized thalli [71]. Additionally, Rorrer [72] also reported that, in order to cultivate marine alga suspension successfully, culture conditions must be similar to those of the parent seaweeds, as demonstrated in the developed phototropic suspension cultures of the brown alga *Laminaria Saccharina* [72]. Isolation and culture of microscopic gametophytes, initiation and culture of callus-like tissue, partial regeneration of freely suspended micro-plantlets from callus-like tissue, and isolation and suspension culture of apical meristems from marine algae are among the techniques used [72]. Suspension culture of *Agardhiella subulata*, a macrophytic red alga, has been reported by Rorrer [72] using two culture steps: (1) undifferentiated filament clumps and (2) micro-plantlets from partial regeneration of the filament clumps. Both cultures were suspended freely in liquid media and can be maintained indefinitely with periodic subcultures. Micro-plantlet suspension cultures were not only used to establish new cultures, but they were also shown to selectively produce bioactive metabolites in *Ochtodes secundiramae*, a macrophytic red alga [73,74].

Schnell et al. [64] described the process of preparing lectin by culturing *Dolichus biflorus* callus in liquid media as a cell suspension culture. The callus tissue grew rapidly, with cell mass increasing by more than 14-fold in just one week. However, after being transferred to new media, the growth decelerated for three days before entering an exponential growth phase for six days before beginning to decline [64]. When compared to lectin production in plants tissue, the amount of lectin production in the plant suspension cultures follows a similar pattern [64]. In marine algae, although the cellular organization of red and brown algae differs, their callus exhibits similar morphology. Their filamentous callus is rigid and was observed to regenerate rapidly into full plants when transferred to liquid media, limiting the range of applications that cell suspension cultures offer in comparison to higher plant applications [63]. While conventional algal lectin extraction methods generally produce higher yields, they do not ensure long-term sustainability of the marine algae because the biomass degrades after a single extraction. Ideally, the production of lectins using a non-destructive extracellular extraction method from cell suspension media would result in more stable and consistent yields. Additionally, Chen et al. [71] predicted that large-scale cell suspension cultures could be developed to produce specific fine chemicals. Rorrer et al. [70] confirmed the finding by successfully developing a photolithothropic suspension culture system of marine algae and biosynthesizing three bioactive hydroxy fatty acids with it. Recently, Polzin and Rorrer [73] successfully cultured *Ochtodes secundiramea* for the production of β-myrcene using micro-plantlet suspension cultures. Reports of higher plants producing lectin in plant cell suspension cultures also hint at the possibility of algal lectin production in suspension cultures [64,65,66,67,68]. Overall, these findings show that lectin can be extracted from marine algae cell suspension culture if the production process is followed correctly.

## 5. Recent Advances in Algal Lectin Isolation

The protein digestibility of marine algae in their raw and unprocessed forms is poor. Therefore, much emphasis has been placed on developing improved methods for algal protein extraction to increase bioavailability [8]. Some of the methods developed by researchers to obtain active compounds from algal biomass without losing their bioactivity are microwave-assisted extraction (MAE), ultrasound-assisted extraction (UAE), enzyme-assisted extraction (EAE), supercritical fluid extraction (SFE), and subcritical water extraction (SWE) [14,75,76]. However, until recently, when Djabayan-Djibeyan et al. [13] developed a novel cold steeping infusion (CSI) technique based on their observations of lectin release from marine algae into their extracellular environment, lectin extraction from marine algae remained limited.

Microwave-assisted extraction (MAE) is a type of microwave heating caused by dipole rotation of a polar solvent and ionic conduction of dissolved ions, which causes cell rupture and compound release into the solvent [77]. MAE reduces solvent consumption and shortens extraction time, making it an efficient technique [58,78]. Ultrasound-assisted extraction (UAE) is typically performed with an ultrasonic bath and ultrasonic probe, in which a solid matrix is dispersed into a solvent in an ultrasonic bath in a stainless steel tank connected to the transducer. UAE improves the extraction process by reducing particle size and increasing mass transfer through the cell wall, which is accomplished through cavitation-induced bubble collapse [79]. It has also been reported that ultrasound pre-treatment increased the protein extraction yield of *Ascophyllum nodosum* by 27–540% and reduced the processing time by 50 min [60]. Meanwhile, enzyme-assisted extraction (EAE) uses enzymes to improve compound extraction in their native form [10], where the hydrolytic actions of enzymes are used to break down the cell walls and cuticles of marine algae. The breakdown is required because the cell walls and cuticles are made up of chemically complex and heterogenous biomolecules that must be broken down to extract bioactive compounds from the environment [10,80]. In supercritical fluid extraction (SFE), a supercritical fluid is a fluid with temperatures and pressures above its critical limit. The density of liquid in this state is similar to that of liquid, while its viscosity is similar to that of gas, and it has intermediate diffusivity between those of gas and liquid. Therefore, supercritical fluids possess better transport properties than liquids and allow for the extraction of bioactive compounds without degradation or loss of volatility [80,81]. Subcritical water extraction (SWE) is typically performed at high temperatures and pressures ranging from 50–200 °C and 50–300 psi for a short period of time with a small amount of solvent [82]. The high pressure causes the solvents to rise above their boiling point, and the increased temperature speeds up the extraction by increasing solubility and mass transfer rate [80,82]. Table 3 lists the various extraction methods used to extract specific compounds in marine algae. Although there has been no specific report on lectin isolation using these methods, the high rate and yield of seaweed protein extraction allows for possible lectin isolation from the crude protein extract. Following that, additional optimization and purification may be required to ensure successful lectin isolation using these assisted methods.

Several studies have shown that marine algae contain lectins capable of producing distinct biological activities such as bacterial, yeasts, and algal aggregation [44], implying that lectins can act as a protection against potentially harmful microorganisms [44,94,95]. Studies have also discovered that marine algae release lectins and lectin inhibitors, such as the monosaccharide *N*-acetyl-D-galactosamine in some algae, into their extracellular environment via a cellular transport system, indicating the presence of lectin compounds in the surrounding media and potentially allowing for lectin recovery from the media [95,96]. Djabayan-Djibeyan et al. [13] developed the cold steeping infusion (CSI) technique in response to their prior study in 2010 [95], which investigated the in vivo lectin release from *Ulva fasciata.* The CSI technique is a novel method for recovering lectins from an algal culture in media without damaging the producing tissues. Even after being activated from dormancy, marine algae can release extracellular bioactive compounds such as lectins, which can then be continuously retrieved from buffer solutions, and the producing algae can be used for subsequent culture cycles.

The CSI technique allows the marine algae to return to its metabolic state after being stored for an extended period of time by immersing it in a simple buffer under an artificial light source. Marine algae could be immersed in a phosphate buffered saline (PBS) solution for 48 h before being collected and treated with solid ammonium sulfate to achieve an 85% sulfate saturation. The crude protein would be released into the PBS solution before being purified further using affinity chromatography [13]. The CSI technique demonstrated similar efficiency in lectin production from the green alga *Caulerpa serrulate* with conventional liquid nitrogen grinding (GLN) method, yielding approximately 407.2 mg of total soluble protein and GLN method yielding 308.4 mg of total soluble protein. After purification with a GalNAc-sepharose affinity chromatography column, the protein attached to the column for the GLN and CSI methods was 4.7 mg and 4.2 mg, respectively. Further analysis using SDS-PAGE and size exclusion chromatography also revealed that lectin isolated using both techniques had similar molecular size and molecular weight. This result showed that the CSI technique does not disrupt lectin structure because the lectin released into the buffer exhibited characteristics comparable to lectin extracted from marine algal tissues.

## 6. Non-Destructive Extracellular Lectin Production in Bioreactors

Seaweed bioprocess engineering is one of the most cutting-edge developments in seaweed biotechnology. Previous research has revealed that seaweeds can be produced and recovered directly from cells and cells aggregates in photobioreactors [97]. Transferring a biosynthetic process from a shake flask to a bioreactor may appear to be simple and straightforward, but there are a few disadvantages in the case of plant cell cultures. The issues may arise as a result of the biosynthesis of chemicals being dependent on several interconnected factors, including compound sensitivity to shear forces, tissue growth rate, plant cell aggregation and wall-associated growth, and cell flotations [73,97,98,99].

**Figure 1:**
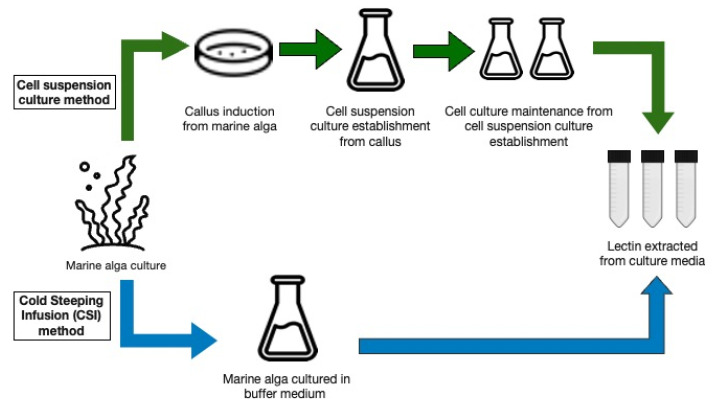
A basic illustration of cell suspension culture and cold steeping infusion (CSI) method for producing lectin in culture media.

The recently developed CSI technique has the potential to be widely used, because it does not require the establishment of cell suspension culture prior to lectin production [13]. As illustrated in Figure 1, this method eliminates the need for suspension culture establishment and a lengthy culture cycle to achieve cell stability for continuous culture.

According to Rao and Ravishankar [100], in vitro cell culture has the advantages of producing bioactive compounds with a defined system and a short culture time, as well as a constant supply of target metabolites. In vitro cultures are often protected from diseases and seasonal variations [101]. On the other hand, the CSI method enables marine algae to revert to a metabolically active state after prolonged storage in buffer solution without causing tissue damage. As a result, this technique is ideal for continuous lectin production since it does not destroy existing algae and allows them to remain as a whole living plant.

The use of a bioreactor to produce plant bioactive compounds allows for better monitoring of the physical and cultural environments, and easier optimization and observation. Yields would be more reproducible and bioactive compound concentrations would be more accessible under controlled growth conditions [72]. Bioprocess technology for marine algae involves the production of cell and tissue cultures, the design of photobioreactors, and the identification of strategies for eliciting secondary metabolite biosynthesis [1]. The growth of marine algae in the bioreactor needs to be optimized to ensure a high yield of lectin production. Marine algae growth and development are influenced by light, temperature, nutrients, salinity, and water movement in the culture media [102].

According to Rorrer and Cheney [1], the airlift photobioreactor shows promising results in terms of mixing, aeration and gas exchange, light transfer, and being less damaging to algal tissues. To integrate the photobioreactor into the CSI system, it could be connected to a chiller and have a jacketed vessel, or it could be kept in a cold room to keep the buffer solution at 4 °C. Maintaining the pH of the buffer solution is also crucial because pH fluctuations can interfere with lectin activity, such as hemagglutination. Additionally, the most important prerequisites for commercial lectin mass production are the selection of a starting material with high lectin production capability and the use of a simple purification technique [3]. *Pterocladiella capillacea* and *Spirogyra* spp. lectins were successfully purified using a cross-linked guar-gum affinity column [103,104], while *Caulerpa serraluta* lectins were purified using a Sepharose-GalNac affinity column [13], *S. filiformis* lectins were purified using a DEAE-sephacel ion exchange chromatography column [32], and *Kappaphycus alvarezii* lectins were purified using an ion exchange chromatography [84]. According to Rorrer and Cheney [1], photobioreactor configuration is related to several effects in culture, including mixing and biomass suspension, aeration and gas exchange, light transfer, and shear damage potential. These factors are critical because they influence the process and end products of the culture. The available photobioreactors include the bubble column photobioreactor, the airlift photobioreactor, the stirred tank photobioreactor (externally illuminated) and the tubular recycle photobioreactor (helical array). Among all photobioreactors, the stirred tank bioreactor demonstrated excellent mixing and biomass suspension, and good to excellent aeration and gas exchange of the culture. However, its light transfer is poor, posing a high risk of shear damage to the culture. On the other hand, an airlift photobioreactor provides very good mixing and biomass suspension quality, as well as better light transfer and a low potential for shear damage in the culture [1]. This photobioreactor would be an excellent candidate for the CSI method to be combined with because it is less likely to damage marine algae in the culture and provides good light transfer to maximize algal productivity.

## 7. Prospects for Algal Lectin Production

Algal lectin’s potential has become more apparent over time as more research has been conducted to demonstrate its benefits. Apart from its antiviral properties, algal lectin has also shown promising results in therapeutic applications. These applications include cancer diagnosis and prognosis, pathological markers of disease, cell–cell communication, and glycan profiling due to its sugar binding specificity for glycoconjugates. As more lectins are extracted and research on the isolated lectins is conducted, it has been discovered that lectin production from marine algae can be improved for maximum benefit.

Significant research on lectin extraction has been conducted in recent decades, and several methods have clearly demonstrated higher lectin yields. Generally, the mechanical method was used, followed by treatment with buffer media and protein precipitation (Table 2). The advancement of various assisted-extraction methods such as MAE, UAE, SFE, and EAE has resulted in a significant increase in the yield of protein extracted while saving more energy and time. Nevertheless, despite the potential improvements made, ongoing research is required to produce lectin on a larger scale and in a more sustainable manner.

Lectin production in culture media would save time and pose fewer health risks to humans and the environment because no harmful solvents would be used in the extraction process. Isolation of lectin from culture media enables the marine algae to be re-cultured and restored to its metabolic functions, preventing the marine algae from being replenished after a single extraction. This feature is highly beneficial to sustainability, because the biomass can be reused for lectin production while also reducing competition with the food industry, where many algae species are primarily grown for food. Therefore, it is crucial to continue the research done by Djabayan-Djibeyan et al. [13,95]. Moreover, additional research in bioreactor systems for marine algal metabolites production will add more insight to the previous discovery [1,72], as many species of marine algae show potential for large-scale cultivation. Large-scale algal lectin production will greatly expand opportunities for algal lectin research, such as antiviral activity against HIV and various influenza viruses. This effort may result in a greater number of therapeutic agents being developed against new viruses that may emerge in the future, as new vaccines may not be readily available at the time.

Inadequate information regarding large-scale cultivation and production of lectin has prevented researchers from conducting commercial trials, as a significantly large amount of marine algae would be needed to run the trial, as opposed to extracting lectin using conventional method, which only uses a smaller amount of marine algae. The large amount of marine algae required in commercial lectin production makes it less appealing, and it is necessary to develop successful culturing technology for targeted lectin production to be more feasible and economically viable. Bioproducts derived from marine algae vary according to species and affected by physical and environmental factors. These factors include nutrient availability, temperature, pH, salinity, light intensity, and carbon dioxide level, as well as the stirring and mixing conditions of the photobioreactor. These factors are vital and must be optimized before conducting a commercial trial. Additionally, commercial lectin production necessitates a fast-growing culture to ensure a higher yield of lectin, such as the supply of some strongly limiting substances or the use of specific growth-promoting bacteria to enhance the growth rate of marine algae.

As interest in marine algae biotechnology grows, more relevant techniques and methods are being developed, and extensive research is required to develop the non-disruptive lectin production method into one of the commercially viable methods. Despite no satisfactory progress in large-scale production of marine algal lectin having yet been reported, the outlook for the near future is optimistic.

## 8. Summary

Marine algae are a rich source of novel lectins. As previously reported, these novel lectins appear to demonstrate antiviral, anticancer, and anti-inflammatory properties, implying a broad range of therapeutic applications. The advancement of lectin extraction techniques from marine algae also aids in the isolation of a greater number of novel lectins. Though these techniques show promising results in lectin extraction at the laboratory scale, more research is needed before they can be applied on a larger scale, such as in a bioreactor or in a commercial setting. Phototrophic suspension culture and micro-plantlet suspension may differ in bioreactor due to their sensitivity to shear force, growth rate and aggregation tendencies. The recently developed CSI technique may be able to overcome these challenges in continuous lectin production from marine algae without causing tissues disruption. This breakthrough is due to the fact that no preceding cell suspension culture is required, and the culture relies on the release of lectin into the culture media by marine algae to ensure continuous recovery. The development of the CSI method is important to reduce the competition of marine algae culture dependence with the food and feed industries. The CSI technology may be able to fulfil the demand for bioactive chemicals derived from marine algae, such as lectin production for future medical and biotechnological applications.

## Figures and Tables

**Table 1:** Protein composition extracted from various marine algae on a dry matter basis (DMB).

Species	Protein Content (Dry Matter Basis)	Reference
*Laminaria hyperborea* ^2^	50.2 ± 2.8	[18]
*Laminaria digitata* ^1^	15.9	[19]
*Ulva lactuca* ^2^	86.5 ± 3.3	[18]
*Ulva rigida* ^2^	112.0 ± 5.8	[20]
*Ulva rotundata* ^2^	100.1 ± 4.9	[20]
*Palmaria palmata* ^2^	122.6 ± 3.1	[18]
*Kappaphycus* sp. ^2^	25−38	[21]
*Porphyra* spp. ^2^	429.9	[22]
*Porphyra acanthophora* ^1^	16.5	[23]

^1^ The figures are in % (*w*⁄*w*) dry weight. ^2^ The figures are in g/kg dry weight.

**Table 2:** Marine algae species and their lectins and specificity, extraction, and purification methods, and reported applications.

Species	Lectin	Specificity	Extraction	Purification	Applications	Reference
*Eucheuma serra*	ESA	Mannose	Phosphate buffer	Ethanol precipitation, fast protein liquid chromatography (FPLC)	Anticancer (apoptosis on cancer cell lines such as OST, LM8, Colo201 and HeLa); antibacterial	[31,33,34,43,44]
*Solieria filiformis*	SfL-1 SfL-2	Mannose	Grinding with liquid nitrogen, phosphate buffer	Ammonium sulfate precipitation, ion-exchange chromatography	Anticancer (apoptosis on cell lines Colo201, LM8 and mouse colon26 adenocarcinoma); induce Th2 immune responses in mouse splenocytes; anti-depressant	[32,43,45,46,47]
*Amansia multifida*	AmL	Avidin, fetuin, mannose	Grinding with liquid nitrogen, sodium phosphate	Ammonium sulfate precipitation, ion-exchange chromatography	Anti-inflammatory action (reducing edema formation, leukocyte migration, and reducing level of proinflammatory cytokines)	[46,48]
*Kappaphycus alvarezii*	KAA-2	High mannose glycan	Homogenization, ethanol	Ethanol precipitation, size exclusion chromatography, ion exchange chromatography	Anti-influenza (inhibits influenza virus propagation by directly binding to high mannose glycans on the envelope glycoprotein hemagglutinin)	[33,49]
*Griffithsia* sp.	Griffithsin	Mannose	Freeze drying, distilled water	Ammonium sulfate precipitation, hydrophobic interaction chromatography	Antiviral (targeting high mannose arrays present on pathogenic enveloped virus such as HIV, coronaviruses, hepatitis C viruses and Japanese encephalitis virus	[35,37,50,51,52,53,54]
*Bryothamnion triquetrum* *Bryothamnion seaforthii*	BTL BSL	Mucins	Grinding with liquid nitrogen, sodium phosphate	Ammonium sulfate precipitation, ion exchange chromatography	Cancer biomarkers, drug delivery	[55,56,57]

**Table 3:** The assisted extraction methods for marine algae and their extracted compounds.

Marine Alga Species	Extraction Method	Isolated Compounds	References
*Ascophyllym nodosum*	Microwave-assisted extraction, ultrasound-assisted extraction	Fucose-sulfated polysaccharides	[9]
*Sargassum aquifolium, Sargassum ilicifolium, Sargassum polycystum*	Enzyme-assisted extraction	Phenolic compound	[11]
*Fucus vesiculosus*	Microwave-assisted extraction	Phlorotannins Polysaccharides	[12]
* Ascophyllum nodosum, Laminaria japonica, Lessonia trabeculate, Lessonia nigrescens *	Microwave-assisted extraction	Phenolic compounds	[77]
*Solieria chordalis*	Microwave-assisted extraction	Carrageenan	[83]
*Ulva pertusa*	Microwave-assisted extraction	Polysaccharides	[84]
* Padina pavonica *	Pressurized liquid extraction, Microwave-assisted extraction	Water extract	[85]
*Pelvetia canaliculate*	Ultrasound-assisted extraction	Antioxidants	[86]
*Nizamuddinia zanardinii*	Ultrasound-assisted extraction	Fucoidans	[87]
*Undaria pinnatifida*	Enzyme-assisted extraction	Fucoxanthin	[88]
*Haematococcus pluvialis*	Supercritical fluid extraction	Astaxanthin	[89]
*Sargassum muticum*	Supercritical fluid extraction	Polyphenols	[90]
*Polysiphonia nigrescens*, *Ulva clathrata*, *Cladophora* sp.	Supercritical fluid extraction with CO_2_	Auxins Cytokinins Polyphenols Microelements Macroelements	[91]
*Saccharina japonica*	Subcritical water extraction	Fucoidan	[92]
*Ascophyllum nodosum*, *Pelvetia canaliculata*, *Fucus spiralis*, *Ulva intestinalis*	Subcritical water extraction	Polyphenols	[93]

## Data Availability

Not applicable.
